# Prediction of posterior elevation stability in keratoconus

**DOI:** 10.3389/fbioe.2023.1288134

**Published:** 2023-11-09

**Authors:** Xiaosong Han, Yang Shen, Dantong Gu, Xiaoyu Zhang, Ling Sun, Zhi Chen, Xingtao Zhou

**Affiliations:** ^1^ Eye Institute and Department of Ophthalmology, Eye and ENT Hospital, Fudan University, Shanghai, China; ^2^ NHC Key Laboratory of Myopia, Key Laboratory of Myopia, Fudan University, Chinese Academy of Medical Sciences, Shanghai, China; ^3^ Shanghai Research Center of Ophthalmology and Optometry, Shanghai, China; ^4^ Shanghai Engineering Research Center of Laser and Autostereoscopic 3D for Vision Care (20DZ2255000), Shanghai, China; ^5^ Clinical Research and Achievement Translation Center, Eye and ENT Hospital, Fudan University, Shanghai, China

**Keywords:** cornea, keratoconus, posterior elevation, corneal topography, Pentacam, machine learning

## Abstract

**Purpose:** This study aimed to investigate the features of progressive keratoconus by means of machine learning.

**Methods:** In total, 163 eyes from 127 patients with at least 3 examination records were enrolled in this study. Pentacam HR was used to measure corneal topography. Steepest meridian keratometry (K_1_), flattest meridian keratometry (K_2_), steepest anterior keratometry (K_max_), central corneal thickness (CCT), thinnest corneal thickness (TCT), anterior radius of cornea (ARC), posterior elevation (PE), index of surface variation (ISV), and index of height deviation (IHD) were input for analysis. Support vector machine (SVM) and logistic regression analysis were applied to construct prediction models.

**Results:** Age, PE, and IHD showed statistically significant differences as the follow-up period extended. K_2_, PE, and ARC were selected for model construction. Logistic regression analysis presented a mean area under the curve (AUC) score of 0.780, while SVM presented a mean AUC of 0.659. The prediction sensitivity of SVM was 52.9%, and specificity was 79.0%.

**Conclusion:** It is feasible to use machine learning to predict the progression and prognosis of keratoconus. Posterior elevation exhibits a sensitive prediction effect.

## Introduction

Keratoconus is a corneal disease featuring progressive ectasia and thinning and typically occurs in teenagers. Aggravating irregular corneal astigmatism can lead to vision loss and compromise of life quality with the progression of keratoconus (Ferdi et al., 2019). It is one of the major causes of blindness.

The effects of various interventions, including rigid gas-permeable contact lenses (RGP), intrastromal corneal rings, corneal cross-linking (CXL), and corneal graft, have been broadly investigated over the years. A previous study indicated a remarkable improvement in best-corrected visual acuity (BCVA) in patients who wear RGP on a long-term basis, while they show no influence on the progression of keratoconus (Araki et al., 2021). Intrastromal corneal rings were shown to profoundly promote visual quality but bring in glare and night vision loss at the same time (Daxer et al., 2010). Not only did CXL surgery improve visual quality and prove its safety, but slowed the progression of keratoconus as well (Hashemi et al., 2013; Hersh et al., 2017; Wang et al., 2018). The effect of CXL with epithelial removal using transepithelial phototherapeutic keratectomy (t-PTK) appeared most notable above all (Kymionis et al., 2012).

Different parameters have been used to evaluate the progression of keratoconus. Chan et al. pointed out that steeper keratometry and younger age could lead to more radical progression (Chan et al., 2020). Another study indicated longitudinal monitoring of corneal topography as a vital perspective (Choi and Kim, 2012). Moreover, predictors with higher efficiency acquired by formulas have shown a new way forward (Shajari et al., 2019; Kato et al., 2021). Various devices play a vital role in the diagnosis and evaluation of keratoconus. The most commonly used protocol is corneal biomechanics combined with topography, including corneal pachymetry, aberration analysis, and optical coherence tomography (OCT) (Martinez-Abad and Pinero, 2017).

With the development of artificial intelligence, prediction efficiency can now be elevated further. Kato et al. used deep learning to predict the need for CXL and found that pachymetry mapping combined with age worked as ideal indicators (Kato et al., 2021), while Ruiz Hidalgo et al. reached a similar conclusion with machine learning (Ruiz Hidalgo et al., 2016). Al-Timemy et al. reported high accuracy in the detection of keratoconus with a time-friendly hybrid deep-learning model (Al-Timemy et al., 2021). The application of machine learning for keratoconus has shown preliminary results and needs further investigation.

The most severe stage of keratoconus usually appears in the second decade of life in Asia. It is inclined to stabilize in the fourth decade of life (Saini et al., 2004; Duncan et al., 2016). With an early onset, keratoconus raises the common concerns of progression and prognosis. Although several studies have investigated single parameters of keratoconus, a precise prediction model remains to be developed. This study aims to investigate the progression and prognosis of keratoconus via machine learning.

## Materials and methods

### Subjects

This is a retrospective study in compliance with the tenets of the Declaration of Helsinki and the request of the Ethics Committee of the Eye and ENT Hospital of Fudan University (Shanghai, China). Informed consent was obtained from the subjects after an explanation of the nature and possible consequences of the study. In total, 163 eyes from 127 patients with keratoconus were recruited. The inclusion criteria were primary keratoconus with no less than 3 examination records on Pentacam HR in the Eye and ENT Hospital of Fudan University and the interval of each follow-up visit should be no less than 1 month. The exclusion criteria were as follows: ectasia after refractive surgery, corneal perforation, history of corneal collagen crosslinking surgery, and history of systematic diseases and other ocular diseases.

### Parameter measurement

Pentacam HR (Oculus, Wetzlar, Germany) was used to analyze the corneal topography. Based on Scheimpflug rotating technology, it reconstructs the anterior segment and exhibits corneal features. Parameters including steepest meridian keratometry (K_1_), flattest meridian keratometry (K_2_), steepest anterior keratometry (K_max_), central corneal thickness (CCT), thinnest corneal thickness (TCT), anterior radial curvature (ARC), posterior elevation (PE), index of surface variation (ISV), index of height deviation (IHD), and staging were input for analysis.

### Machine learning

Clustering and machine learning were combined to construct a prediction model based on the patients’ group level. A variety of regression algorithms of machine learning including regression trees model (CART), support vector machine (SVM), random forest (RF), and extreme gradient boosting (XGBoost) was conducted. Ten-fold cross-validation was used for model training. Of the data, 70% were randomly chosen for the test set and 30% randomly for the training set. There are 2 sets in 10-fold cross-validation. The training set was created for learning and the test set for validation, which tests the efficiency of models. Performing 10-fold cross-validation is meant to provide a reliable estimate of prediction accuracy, even though it may require longer processing time. The LASSO (Least Absolute Shrinkage and Selection Operator) model was used to select the parameters with the highest performance in SVM and logistic regression, while the RFE (Recursive Feature Elimination) model was used to do the same in CART and RF. The parameters of XGBoost were selected in the model automatically. Mutual parameters shared by at least four models were defined as the parameters with the highest performance.

### Statistical analysis

Statistical analysis was performed using R version 4.1.12. Continuous variables were presented as mean ± standard deviation (SD), and categorical variables were presented as frequency and percentage. Machine learning was processed with Python (3.0).

## Results

In total, 163 eyes from 127 patients (male, 99; female, 28) were enrolled in this study with no corneal infection or perforation. The mean age of diagnosis was 20.1 ± 5.4 years old, ranging from 7 to 34 years old. The mean follow-up time was 55.50 ± 27.75 months.

**FIGURE 1:**
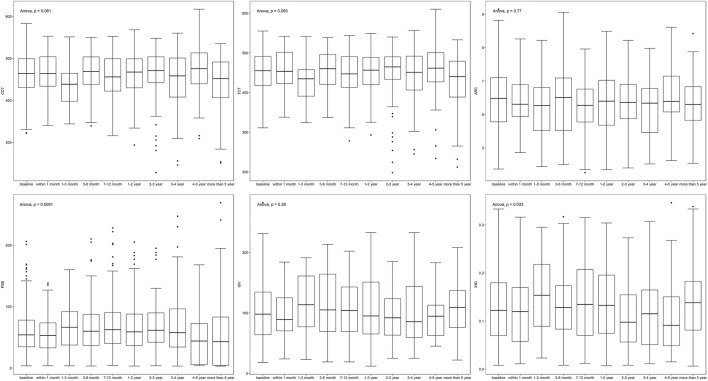
Changes of parameters during follow-ups.

Parameters fluctuated during follow-up (Table 1; Figure 1). K1, K2, and Kmax showed a down-up-down trend, while CCT and TCT shared the same trend. ISV and IHD varied similarly and simultaneously. The changes in age (*p* < 0.001), PE (*p* = 0.009), and IHD (*p* = 0.033) were statistically significantly different.

**TABLE 1:** Parameters in different stages of follow-up.

	Baseline	Within 1 month	1–3 months	3–6 months	7–12 months	1–2 years	2–3 years	3–4 years	4–5 years	More than 5 years	*p*
n	163	34	42	63	102	130	90	58	44	91	
age	20.05 ± 5.39	21.10 ± 5.57	21.33 ± 5.82	20.18 ± 4.81	20.75 ± 5.22	21.40 ± 5.69	22.14 ± 5.40	23.03 ± 4.94	25.02 ± 4.98	25.77 ± 4.62	<0.001
K_1_	48.25 ± 6.55	48.70 ± 6.25	49.39 ± 7.86	49.53 ± 7.50	49.31 ± 6.18	48.94 ± 6.52	50.09 ± 7.24	51.59 ± 8.06	49.28 ± 5.45	50.86 ± 6.65	0.109
K_2_	52.11 ± 6.87	52.00 ± 6.35	53.06 ± 8.19	52.59 ± 7.79	52.12 ± 6.47	51.73 ± 6.93	53.54 ± 7.15	54.20 ± 8.23	53.08 ± 5.89	54.24 ± 6.91	0.258
K_max_	59.90 ± 10.88	58.88 ± 10.12	60.50 ± 11.39	60.68 ± 11.79	59.50 ± 10.04	58.39 ± 10.14	59.86 ± 9.68	60.87 ± 11.38	59.40 ± 8.93	61.04 ± 10.10	0.879
CCT	461.45 ± 53.28	466.09 ± 49.89	436.26 ± 47.37	462.87 ± 49.91	456.03 ± 55.39	461.93 ± 51.85	461.71 ± 61.91	451.53 ± 68.49	470.32 ± 58.50	447.12 ± 62.97	0.081
TCT	450.67 ± 54.52	459.29 ± 48.76	428.05 ± 47.08	452.06 ± 48.98	444.50 ± 57.06	449.25 ± 51.46	450.54 ± 68.20	441.12 ± 67.00	456.82 ± 70.07	432.68 ± 67.16	0.093
ARC	6.45 ± 0.93	6.42 ± 0.86	6.29 ± 0.96	6.33 ± 1.04	6.24 ± 0.87	6.35 ± 0.93	6.35 ± 0.82	6.25 ± 0.92	6.50 ± 0.78	6.35 ± 0.85	0.773
PE	61.03 ± 40.37	57.09 ± 34.71	69.73 ± 41.90	70.76 ± 48.35	72.91 ± 47.66	68.31 ± 45.25	69.35 ± 42.46	73.19 ± 58.20	50.99 ± 50.06	50.93 ± 53.55	0.009
ISV	103.29 ± 50.84	95.82 ± 44.26	113.83 ± 48.67	112.16 ± 51.47	107.44 ± 49.93	104.78 ± 52.89	94.48 ± 40.71	101.48 ± 50.53	94.48 ± 38.04	107.87 ± 46.44	0.284
IHD	0.13 ± 0.08	0.13 ± 0.08	0.15 ± 0.08	0.14 ± 0.08	0.14 ± 0.08	0.14 ± 0.08	0.11 ± 0.06	0.12 ± 0.07	0.11 ± 0.08	0.14 ± 0.08	0.033

**FIGURE 2:**
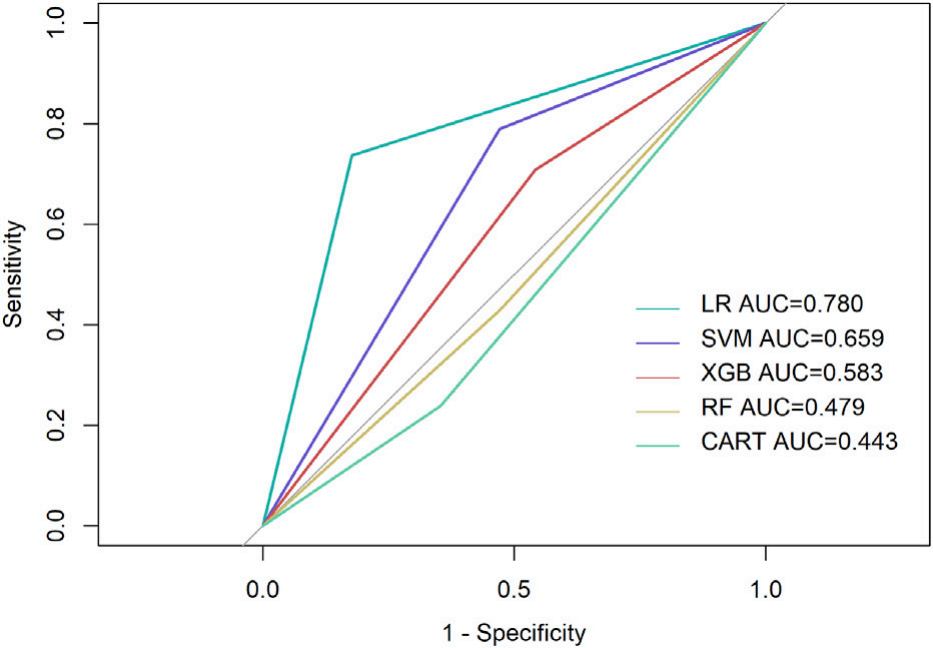
Performance of prediction models. LR, Logistic regression; Purple line, reference line, showing AUC score of 0.500.

Among all the parameters input, K2, PE, and ARC showed the highest performance in most models and were screened for machine learning and logistic regression. The AUC scores of three out of five prediction models were above 0.500 (Table 2), while the logistic regression model presented a higher AUC value of 0.780 (Figure 2), followed by SVM with 0.659. The prediction sensitivity of the logistic regression model reached 0.842, followed by XGBoost (0.640) and CART (0.619), while SVM showed the highest prediction specificity with 0.790, followed by logistic regression (0.737) and RF (0.684).

**TABLE 2:** Comparison of the performance of prediction models.

	SVM	CART	RF	XGBoost	Logistic regression
AUC	0.659	0.443	0.479	0.583	0.780
Sensitivity	0.529	0.619	0.571	0.640	0.824
Specificity	0.790	0.238	0.684	0.520	0.737

## Discussion

Leading to blindness, keratoconus has an overall incidence of 1/2000 (Gomes et al., 2015). While a consensus on diagnosis and treatment has been reached over the past 20 years, the criteria of progression and prognosis remain to be discussed. To our knowledge, this study predicted the development of keratoconus with Pentacam via machine learning for the first time.

It was revealed in our study that changes in age, PE, and IHD showed statistical significance in the progression of keratoconus, which has been proved in previous studies. Serving as indications of elevation, PE shows the variation directly, while IHD does so with a processed index, which implies the indication effect of posterior elevation in the progression and prognosis of keratoconus. Younger age and severity of onset indicate more radical development (Meyer et al., 2021; Ferdi et al., 2022). Moreover, it was implied that a steeper K_max_ is a risk factor for the progression of keratoconus (Hamilton et al., 2016). This could be attributed to the interaction between K_max_ and other parameters such as TCT, which becomes thinner with the steepening of K_max_. While the change is reflected in staging, it fails to indicate the progression of the disease. Age and PE are relatively more sensitive and could serve as early warning parameters.

It has been pointed out that ISV is also a sensitive predictor of the progression of keratoconus (Kanellopoulos and Asimellis, 2013), which could be related to its role in the staging algorithm of Pentacam. It was included in the machine learning input to improve the accuracy of prediction.

The AUC value of the logistic regression model was higher than that of the SVM model. This could be related to the appropriate condition of both algorithms. While the SVM model is a deep learning method that is relatively well adapted to limited samples, a multi-classification task remains a problem. On the contrary, logistic regression analysis is an ideal method for the prediction of complex prognosis, which explains the higher AUC value compared to the SVM model.

The limitations of our study are 1) The sample size is relatively small and 2) the eye-rubbing habits and genetic backgrounds of patients remain to be taken into consideration for better stability of prediction.

In conclusion, posterior elevation serves as a sensitive predictor of the progression of keratoconus. It is feasible to predict the development of keratoconus via machine learning.

## Data Availability

The raw data supporting the conclusion of this article will be made available by the authors, without undue reservation.
